# A powder method for the high-efficacy evaluation of electro-optic crystals

**DOI:** 10.1093/nsr/nwaa104

**Published:** 2020-05-28

**Authors:** Feng Xu, Ge Zhang, Min Luo, Guang Peng, Yu Chen, Tao Yan, Ning Ye

**Affiliations:** Key Laboratory of Optoelectronic Materials Chemistry and Physics, Fujian Institute of Research on the Structure of Matter, Chinese Academy of Sciences, Fuzhou 350002, China; University of the Chinese Academy of Sciences, Beijing 100049, China; Fujian Science & Technology Innovation Laboratory for Optoelectronic Information of China, Fuzhou 350002, China; Key Laboratory of Optoelectronic Materials Chemistry and Physics, Fujian Institute of Research on the Structure of Matter, Chinese Academy of Sciences, Fuzhou 350002, China; Fujian Science & Technology Innovation Laboratory for Optoelectronic Information of China, Fuzhou 350002, China; Key Laboratory of Optoelectronic Materials Chemistry and Physics, Fujian Institute of Research on the Structure of Matter, Chinese Academy of Sciences, Fuzhou 350002, China; Fujian Science & Technology Innovation Laboratory for Optoelectronic Information of China, Fuzhou 350002, China; Key Laboratory of Optoelectronic Materials Chemistry and Physics, Fujian Institute of Research on the Structure of Matter, Chinese Academy of Sciences, Fuzhou 350002, China; Fujian Science & Technology Innovation Laboratory for Optoelectronic Information of China, Fuzhou 350002, China; Key Laboratory of Optoelectronic Materials Chemistry and Physics, Fujian Institute of Research on the Structure of Matter, Chinese Academy of Sciences, Fuzhou 350002, China; Fujian Science & Technology Innovation Laboratory for Optoelectronic Information of China, Fuzhou 350002, China; Key Laboratory of Optoelectronic Materials Chemistry and Physics, Fujian Institute of Research on the Structure of Matter, Chinese Academy of Sciences, Fuzhou 350002, China; Fujian Science & Technology Innovation Laboratory for Optoelectronic Information of China, Fuzhou 350002, China; Key Laboratory of Optoelectronic Materials Chemistry and Physics, Fujian Institute of Research on the Structure of Matter, Chinese Academy of Sciences, Fuzhou 350002, China; Fujian Science & Technology Innovation Laboratory for Optoelectronic Information of China, Fuzhou 350002, China

**Keywords:** electro-optic crystals, powder crystals, high-efficacy evaluation method, electro-optic coefficients

## Abstract

The electro-optic crystal holds great promise for extensive applications in optoelectronics and optical communication. However, the discovery of novel electro-optic crystals is sporadic due to the difficulties of large-sized crystal growth for electro-optic coefficient measurement. Herein, to address this issue, a high-efficacy evaluation method using accessible powder samples is proposed in which the second-harmonic-generation effect, infrared reflectance spectrum and Raman spectrum are introduced to predict the magnitude of the electro-optic coefficient. The calculated electro-optic coefficients of numerous reported electro-optic crystals through this approach give universal agreement to the experimental values, evidencing the validity of the strategy. Based on this method, CsLiMoO_4_ is screened as a novel potential electro-optic crystal and a high-quality crystal is grown by the Czochralski technique for electro-optic coefficient measurement using the half-wave voltage method, the result of which is also comparable to the calculated value. Consequently, the evaluation strategy presented here will pave a new way to explore promising electro-optic crystals efficiently.

## Teaser

A high-efficacy powder method, combining calculations and measurements on second harmonic generation effect, infrared reflectance spectrum, and Raman spectrum, is proposed for the evaluation and exploration of novel electro-optic crystals.

## INTRODUCTION

In recent years, the electro-optic (E-O) crystal has shown great potential for broad applications including an E-O switch, a high-speed E-O modulator, a deflector and laser mode-locking. Particularly, with the prosperous development of the Terahertz (THz) spectroscopy technique, E-O crystals have been employed in this realm for the generation and detection of THz electromagnetic radiation [1–3]. Although there are some commercial E-O crystals available in the marketplace, further exploration of novel E-O crystals with superior properties is also in great demand for a variety of current applications.

While extensive research on the E-O crystal was carried out in the 1960s [4], the exploration of novel E-O crystals still progresses slowly. Nowadays, the screening of E-O crystals mainly concentrates on those known non-linear optical (NLO) crystals owing to their same prerequisite of belonging to non-centrosymmetry (NCS) point groups [5]. Many familiar E-O crystals, such as KH_2_PO_4_ (KDP) [6,7], NH_4_H_2_PO_4_ (ADP) [6,7], KD_2_PO_4_ (DKDP) [7–9], LiNbO_3_ [10,11], LiTaO_3_ [9,12], RbTiOPO_4_ (RTP) [13], KTiOPO_4_ (KTP) [13,14] and La_3_Ga_5_SiO_14_ (LGS) [15], were identified in this way. For instance, as a superior NLO crystal with a broad phase-matchable range, large effective SHG coefficient, high damage threshold and thermal stability, the β-BBO crystal [16], crystallized in NCS point group 3m, was considered a candidate E-O crystal [17,18]. To assess the E-O property of the β-BBO crystal, E-O coefficients, as the most significant factor of the E-O effect, should be acquired, which were usually measured through a millimeter-sized single crystal. Nevertheless, growing high-quality crystals with large size for E-O coefficient measurement was another challenge. After many efforts, the success of large-sized crystal growth and the measurement of E-O coefficients in recent years have rendered the β-BBO crystal to be a practical E-O crystal, extensively applied in the high-average-power E-O Q-Switch [19]. Although this evaluation strategy was favorable for exploring novel E-O crystals, it was inefficient due to the employment of large-sized crystals. Furthermore, it was worth noting that some point groups were excluded during the discovery of NLO crystals such as 422, 622, 23 and $\bar{4}3 {\rm m}$, taking into consideration the requirement for Kleinman symmetry and refractive-index anisotropy for phase-matching in a cubic system. As a result, a number of crystals were neglected inherently when seeking potential E-O crystals from NLO crystals. This unsystematic and inefficient method limited the exploration of E-O crystals to a large extent. Hence, the evaluation method for E-O crystals should be updated urgently.

To improve the evaluation strategy of E-O crystals, many efforts were made to reveal the essence of the E-O effect. Kurtz and Robinson presented a physical model of the E-O effect based on an extension of Bloembergen's anharmonic oscillator model for NLO processes [4]. Faust and Flytzanis utilized the classic harmonic-oscillator model and the electrostatic point-charge model to attest to the physical essence of the E-O effect [20,21]. These results indicated the significant contribution of the lattice vibration to the E-O effect. Fousek utilized a classical harmonic oscillator and macroscopical structure of lattice variation to analyse the change in refractive indices induced by structural phase transitions and to illustrate the E-O effect [22]. But it was only a qualitative description through the analyses of physical images and cannot explain the E-O effect in detail for most materials. In 1982, a simple theoretical study of the linear E-O effect, based on the single-energy-gap model, dielectric theory and the concepts of bond charge and effective ionic charge, was presented by Yariv [23]. This approach can provide an expression for the E-O coefficient and was applied to some diatomic and ternary compounds but not to complex crystals.

Inspired by the previous work mentioned above, we attempted to build a new evaluation method to predict E-O coefficients efficiently, in which the lattice vibration and charge movements were considered to be the determining factors of the E-O effect microscopically. Generally, the first-principle calculation was one of the most suitable keys to analyse the lattice vibration and charge movements for most materials and was effective in evaluating the electro-optic effect of a known crystal [24,25]. However, the corresponding first-principle calculation was generally too complicated for many experimental scientists. Besides, comparing high-quality large-sized crystals employed for E-O coefficients measurement, only powders or small crystals of micron size were easy to be synthesized but not large enough for evaluating the E-O coefficient using only an experimental technique (e.g. the half-wave voltage method). In view of this, herein, a powder method, combining theoretical calculation and experimental technique to analyse the lattice vibration and charge movements of materials instead of the first-principle calculation, was developed constructively to obtain the E-O coefficients of materials efficiently. The calculated E-O coefficients of known E-O crystals through this powder method were studied, showing universal agreement with the experimental values. Furthermore, on the strength of this method, the calculated E-O coefficient of CsLiMoO_4_ (CLM) was also obtained, which matched well with the measured value using high-quality millimeter-sized CLM crystal grown by the Czochralski technique, demonstrating it to be a novel potential E-O crystal. Significantly, the powder method proposed here shows great promise for the high-efficacy evaluation of E-O crystals.

## THEORETICAL CALCULATION

This work concentrated on the linear E-O effects of crystals because the linear part of most materials was generally more prominent than the quadratic one. Since the E-O effect was derived from the contributions of charge movements and the lattice vibration, the linear E-O coefficient was expressed by:

(1) \begin{equation*}\gamma = {\gamma ^e} + {\gamma ^o} + {\gamma ^a},\end{equation*}

where *γ^e^* denotes the contribution of charge movements; *γ^o^* and *γ^a^* represent the contributions of the optical modes and acoustic modes of lattice vibration, respectively. The acoustic modes of lattice vibration can be ignored here because they contributed little to the linear E-O coefficients for most of the E-O materials. In this work, the investigations of the contribution of charge movements were carried out through powder SHG measurements. Experimentally, since the optical modes of lattice vibration were characterized by the Raman scattering efficiency and infrared oscillator strength [26–29], we attempted to employ the infrared reflectance spectrum (IRRS) and Raman spectrum to describe the contribution of lattice vibration to materials in powder form. Generally, the E-O coefficient *γ* was related to the linear E-O susceptibility *χ*^(2)^(–*ω*, *ω*, 0). Further, the contribution of the lattice vibration of *χ*^(2)^(–*ω*, *ω*, 0) corresponded to the linear susceptibility *χ*^(1)^(*ω*) and transition probability of Raman scattering *W*, which can be obtained from the IRRS and Raman spectrum, respectively. Here, we defined *M*(*r*) and *P*(*r*) as the dipole transition matrix element and the transition-susceptibility matrix element, which were associated with *χ*^(1)^(*ω*) and *W*, respectively. These two matrix elements can be expressed as follows [30–32]:

(2) \begin{equation*}{\chi ^{(1)}}(\omega ) = f\!(\omega )\frac{1}{\hbar }\sum\limits_i {\frac{{2{\omega _i}}}{{\omega _i^2 - {\omega ^2} - i\omega {y_i}}}{M^2}(r)} ,\end{equation*}

(3) \begin{equation*}W(r) = {f^2}({\omega _1}){f^2}({\omega _2})\frac{{\sqrt {{\varepsilon _2}} {\omega _1}\omega _2^3}}{{{\varepsilon _1}{\hbar ^2}{c^3}}}N{P^2}(r).\end{equation*}

**Figure 1.**
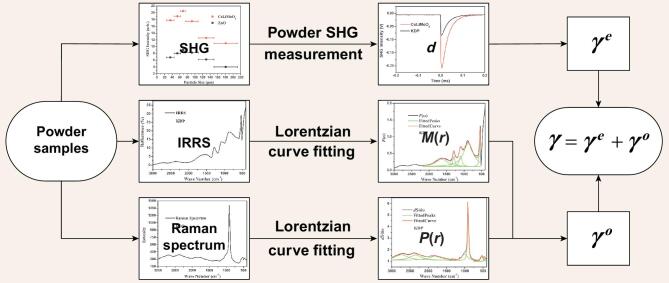
Schematic illustration of the powder method using powder SHG measurement, IRRS and Raman spectrum.

In Equation (2), *f*(*ω*) denotes the local field correlation; *ω_i_* and *y_i_* represent each center frequency and the peak width of the relevant fitted peaks, respectively. In Equation (3), *N* denotes the number of particles; *ω*_1_ and *ω*_2_ represent the incident light frequency and scattered light frequency of the Raman scattering. The computation process of our powder method is illustrated in Fig. 1 and detailed in the following.

From the IRRS, the relationship between reflectance *R*(*k*) and wave numbers *k* can be acquired. The complex amplitude reflectance $\hat{r}$ and phase shifts $\phi (k)$ are expressed in terms of the Kramers–Kronig relation as:

(4) \begin{equation*}\hat{r} = {R^{1/2}}{e^{i\phi }},\end{equation*}

(5) \begin{equation*}\phi (k) = \frac{k}{\pi }\int_{0}^{\infty }{{\frac{{\ln R(K) - \ln R(k)}}{{{k^2} - {K^2}}}d\!K}}.\end{equation*}

Thus, the relationship between the complex amplitude reflectance $\hat{r}$ and reflectance *R*(*k*) can be derived. In this work, the dielectric function and the refractive index, defined as $\hat{\varepsilon }(\omega ) = \varepsilon ^{\prime}(\omega ) + i\varepsilon ^{\prime\prime}(\omega )$ and $\hat{n}(\omega ) = n(\omega ) - i\kappa (\omega )$, respectively, can be described with reflectance *R*(*k*) based on Fresnel's formula $\hat{r} = (\hat{n} - 1)/(\hat{n} + 1)$ and the relation $\hat{\varepsilon } = {\hat{n}^2}$. Besides, the relationship between the local field correlation and the dielectric function are represented as $f\!(\omega ) = [\varepsilon ^{\prime}(\omega ) + 2]/3$ according to the Lorentz model. Consequently, in terms of the linear relation $\hat{\varepsilon } = 1 + 4\pi {\chi ^{(1)}}$, the linear susceptibility *χ*^(1)^(*ω*) in Equation (2) can be derived from the dielectric function, connecting with reflectance *R*(*k*) from the IRRS. Comparing the derived formula to Equation (2), we can define a function *F*(*ω*) as:

(6) \begin{eqnarray*}F\!(\omega ) &=& \frac{{\varepsilon ^{\prime\prime}(\omega )}}{{\varepsilon ^{\prime}(\omega ) + 2}} \nonumber\\ &=& \sum\limits_i {\frac{{8\pi }}{{3\hbar }}\frac{{\omega {\omega _i}{y_i}}}{{{{({\omega ^2} - \omega _i^2)}^2} + {\omega ^2}y_i^2}}{M^2}(r)} .\nonumber\\ \end{eqnarray*}

Significantly, the expression of *F*(*ω*) shows agreement with the Lorentzian curves. The peak height *H_i_* can be derived from Equation (6) as follows:

(7) \begin{equation*}{H_i} = \frac{{8\pi }}{{3\hbar }}\frac{{{M^2}(r)}}{{{y_i}}}.\end{equation*}

Therefore, the Lorentzian curve fitting was introduced in the function *F*(*ω*) to obtain the magnitude of each peak height *H_i_* and peak width *y_i_* of the Lorentzian curves. And the magnitude of *M*(*r*) can be figured out according to Equation (7), contributing to the calculation of E-O coefficients.

On the analysis of the Raman spectrum, the scattering efficiency of Raman scattering *S* was defined as [30,32]:

(8) \begin{equation*}S = \frac{{4W}}{{{{(1 + n)}^2}{N_i}c}},\end{equation*}

where *n* denotes the refractive index of the powder sample. The transition probability of Raman scattering *W* is associated with the transition-susceptibility matrix element *P*(*r*) according to Equation (3). The particle number *N_i_* of the incident light was fitted to the Bose–Einstein distribution, whose expression is ${N_i} = {[\exp (\hbar {\omega _i}/kT) - 1]^{ - 1}}$. Combining Equations (8) and (3), we summarized the relationship between *S* and *P*(*r*) as:

(9) \begin{eqnarray*}\frac{{d\!S}}{{d\omega }} &=& \frac{4}{{9{\hbar ^2}{c^4}}}\frac{{{{(\varepsilon + 2)}^2}n}}{{{{(1 + n)}^2}}}\omega {(\omega - {\omega _i})^3}({N_i} + 1)\nonumber\\ &&\times\,\,\frac{{{y_i}/\pi }}{{{{(\omega - {\omega _i})}^2} + y_i^2}}{P^2}(r),\end{eqnarray*}

where *ω* represents the frequency of the incident light under laser excitation. Inspecting Equation (9) carefully, we found that it was also in accordance with the expression of Lorentzian curves. As such, Lorentzian curve fitting was employed in Equation (9) as follow:

(10) \begin{equation*}\frac{{d\!S}}{{d\omega }} = \sum\limits_i {{H_i}\frac{{y_i^2}}{{{{(\omega - {\omega _i})}^2} + y_i^2}}} ,\end{equation*}

(11) \begin{eqnarray*}{H_i} &=& \frac{4}{{9{\hbar ^2}{c^4}}}\frac{{{{(\varepsilon + 2)}^2}n}}{{{{(1 + n)}^2}}}\omega {(\omega - {\omega _i})^3}\nonumber\\ &&\times\,({N_i} + 1)\frac{{{P^2}(r)}}{{{y_i}}},\end{eqnarray*}

where *ω_i_*, *y_i_* and *H_i_* denote the magnitude of the center frequency, peak width and peak height of each fitted peak, respectively. Since *dS*/*dω* was derived directly from the relative intensity of the Raman scattering, the values of *ω_i_*, *y_i_* and *H_i_* can be obtained expediently from the Raman spectrum through the fitting process. Accordingly, the magnitude of *P*(*r*) was calculated according to Equation (11) using the fitted peak parameters. It should be noted that the modes of lattice vibration expressed in the IRRS and Raman spectrum were distinguished in the calculation process owing to the utilization of powder samples.

Combining the measurements and analyses of powder SHG responses, IRRS and Raman spectrum, the calculated E-O coefficient was given by:

(12) \begin{equation*}\gamma = d + \frac{1}{{\sqrt 8 }}\sum\limits_r {\frac{{M(r)P(r)}}{{{\hbar ^2}{\omega _r}}}},\end{equation*}

where *d* denotes the powder SHG coefficient without local electric field; *M*(*r*) and *P*(*r*) were derived from Equations (7) and (11), respectively. Here, *ω_r_* denotes the corresponding center frequency where the fitted peaks overlapped from functions *F*(*ω*) and *dS*/*dω*. Only the peaks located at the same center frequency to a certain extent contributed to E-O coefficient *γ*. As displayed in Fig. 1, the magnitude of the E-O coefficients can be predicted using the powder method for the evaluation of E-O crystals efficiently, on the basis of our experiments and calculations including powder SHG measurement and the analyses of the IRRS and Raman spectrum.

## CALCULATION AND VERIFICATION OF DISCOVERED E-O CRYSTALS

**Figure 2.**
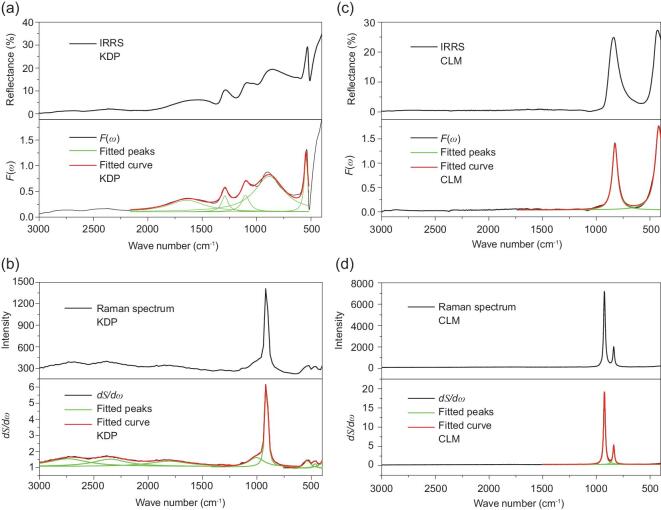
(a) The IRRS and *F*(*ω*), (b) Raman spectrum and *dS*/*dω* of KDP powder. (c) The IRRS and *F*(*ω*), (d) Raman spectrum and *dS*/*dω* of CLM powder.

To certify the validity of the powder method, numerous known E-O materials were selected to calculate their E-O coefficients. The SHG coefficients were consulted from *Non-linearOptical Crystals: A Complete Survey* [33] or measured using the powder SHG method adapted from Kurtz and Perry [34] (shown in the ‘Methods’ section). Taking the practical and commercial E-O crystal KDP as an example, the IRRS and Raman spectrum of KDP powder as well as the curve-fitting results are shown in Fig. 2a and b. According to our powder method, the calculated E-O coefficient of KDP determined using Equation (12) was 10.69 pm V^–^^1^, in agreement with the experimental value of 10.50 pm V^–^^1^ [33]. The detailed calculated process is displayed in the Supplementary data. In addition to that of KDP, the E-O coefficients of other reported E-O crystals were calculated through the proposed powder method and the results are summarized in Table 1. The calculated E-O coefficients of these known E-O materials matched well with the measured values, demonstrating the availability of the powder method.

**Table 1.** The experimental and calculated values of E-O coefficients of known E-O materials.

Crystals	Point group	Wavelength (μm)	Contribution of lattice vibration (pm V^–1^)	Contribution of charge movements (pm V^–1^)	Calculated E-O coefficient (pm V^–1^)	Measured E-O coefficient (pm V^–1^)
KDP	$\bar{4}2{\rm m}$	0.532	10.30	0.39 [33]	10.69	10.50 [33]
DKDP	$\bar{4}2{\rm m}$	0.532	21.65	0.37 [33]	22.02	24.00 [33]
ADP	$\bar{4}2{\rm m}$	0.532	5.12	0.47 [33]	5.59	5.55 [33]
β-BBO	3m	0.532	0.00	2.20 [33]	2.20	2.10 [33]
LiNbO_3_	3m	0.633	14.83	19.50 [33]	34.33	30.80 [33]
LiTaO_3_	3m	0.633	17.95	10.70 [33]	28.65	30.50 [33]
KTP	mm2	0.633	21.05	11.10 [33]	32.15	36.30 [33]
La_3_Ga_5_SiO_14_	32	0.532	1.33	1.70 [35]	3.03	2.69 [15]
La_3_Ga_5.5_Nb_0.5_O_14_	32	0.532	0.69	2.60 [35]	3.29	2.63 [15]
La_3_Ga_5.5_Ta_0.5_O_14_	32	0.532	1.84	2.30 [35]	4.14	2.83 [15]
BaTeMo_2_O_9_	2	0.633	10.97	3.13 [36]	14.10	9.00 [37]
Cs_2_TeMo_3_O_12_	6	0.532	12.99	6.50 [38]	19.49	11.08 [39]

## EXPLORATION ON NOVEL E-O CRYSTALS

Following the evaluation method using powder samples, the approximate magnitude of the E-O coefficients for a few potential E-O crystals were also predicted and are shown in Table 2. As a prospective E-O crystal, CsLiMoO_4_, synthesized in powder form using the conventional solid-state reaction (shown in the ‘Methods’ section), was employed to collect the IRRS and Raman spectrum for evaluating the contribution of lattice vibration (Fig. 2c and d). The PXRD pattern of CLM is shown in Supplementary Fig. 1, which verified the purity of the sample. The calculated value of the contribution of lattice vibration in the E-O coefficient was finally derived as 11.61 pm V^–^^1^ through the proposed powder method. According to the theory of Kurtz and Perry, the powder SHG measurement in different ranges of particle size was performed for CLM. The relationship between particle sizes and SHG responses revealed that the CLM are not phase-matchable at the wavelength of 1064 nm, as shown in Supplementary Fig. 2. As a reference, the ZnO crystal, a familiar non-phase-matchable material, was ground and sieved into the same particle-size ranges for measurements, whose derived effective NLO coefficient *d*_eff_ was ∼0.43 pm V^–1^ [40]. In the particle-size range of 62–75 μm, the SHG ratio of CLM to ZnO was approximately 2.22 (see Supplementary Fig. 2) and the calculated SHG coefficient for CLM was 0.96 pm V^–1^. Therefore, the calculated E-O coefficient for CLM was 12.57 pm V^–^^1^, which was derived from the powder SHG coefficient and the calculated results from the IRRS and Raman spectrum.

**Table 2.** The calculated E-O coefficient of potential E-O crystals.

Crystals	Point group	Wavelength (μm)	Contribution of lattice vibration (pm V^–1^)	Contribution of charge movements (pm V^–1^)	Calculation of E-O coefficient (pm V^–1^)
CsLiMoO_4_	$\bar{4}3{\rm m}$	0.532	11.61	0.96 [this work]	12.57
MgTeMoO_6_	222	0.532	9.17	18.72 [41]	27.89
LiNa_5_Mo_9_O_30_	mm2	0.532	3.32	0.78 [42]	4.10
CsLiWO_4_	$\bar{4}3{\rm m}$	0.532	8.31	0.94 [this work]	9.25
Rb_2_Mg_2_(WO_4_)_3_	23	0.532	5.02	0.12 [43]	5.14
Cs_2_Mg_2_(WO_4_)_3_	23	0.532	7.97	0.12 [43]	8.09
Rb_2_TeW_3_O_12_	3m	0.532	12.05	6.20 [44]	18.25
Cs_2_TeW_3_O_12_	6	0.532	10.12	6.20 [44]	16.32
Li_3_VO_4_	mm2	0.532	13.29	3.71 [45]	17.00
Ba_3_(ZnB_5_O_10_)PO_4_	mm2	0.532	1.43	0.78 [46]	2.21
RbPbBP_2_O_8_	$\bar{4}2{\rm m}$	0.532	9.61	0.39 [47]	10.00
Cs_2_Bi_2_O(Ge_2_O_7_)	mm2	0.532	7.60	1.44 [48]	9.04
Li_2_K_4_[(TiO)Si_4_O_12_]	4mm	0.532	3.94	0.83 [49]	4.77

Among these candidates, CsLiMoO_4_ (CLM) was screened as the preferred one because of the moderate calculated E-O coefficient, which was comparable to that of KDP. Then, the relationship between crystal symmetry and the E-O configurations was required to be considered during crystal growth and device design. In this case, CLM, crystallized in point group $\bar{4}3{\rm m}$, has only one independent and non-vanishing linear E-O coefficient, namely *γ*_41_, which made it easy to realize the transverse configuration of the E-O effect and device design with a low half-wave voltage. Furthermore, the crystal in the $\bar{4}3{\rm m}$ point group was not influenced by the optical rotation and phase difference caused by natural birefringence during the operation process. Besides, the result of thermal analysis (see Supplementary Fig. 3) revealed that CLM melted congruently and can be easily grown to a large size compared with those incongruent potential crystals with a large coefficient.

**Figure 3.**
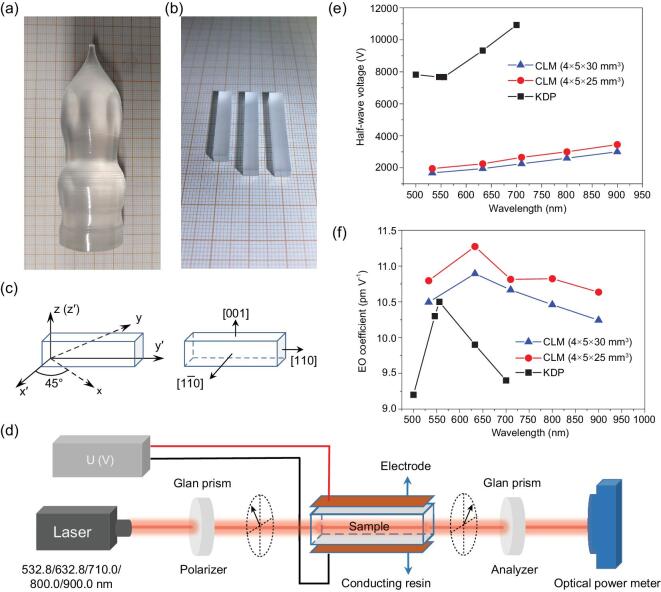
Photographs of (a) CLM crystal and (b) E-O component. (c) The crystal orientation of the E-O component. (d) Experimental configuration for E-O measurement using the half-wave voltage method. (e) The half-wave voltages and (f) E-O coefficients of CLM and KDP at different wavelengths.

To further investigate the E-O property of CLM and evidence the validity of the proposed evaluation method, transparent, core-free and good-quality single crystals of CLM were successfully grown by the Czochralski method and are shown in Fig. 3a [50]. A CLM crystal of size 10 × 10 × 1 mm^3^ was polished for the measurement of refractive indices using the prism-coupling method. The data were recorded at five different monochromatic sources (0.407, 0.532, 0.636, 0.984 and 1.547 μm) (see Supplementary Fig. 4). To obtain the E-O coefficient, the measured refractive indices *n* of CLM as a function of the wavelength were fitted using the least-squares method according to the Sellmeier equations (see Supplementary Fig. 4): *n*^2^ = A + B/(*λ*^2^ – C) – D *λ*^2^, where *λ* is the wavelength in μm and A–D are the parameters. The fitted Sellmeier equation was expressed as

(13) \begin{equation*}{n^2} = 2.46835 + \frac{{0.01797}}{{{\lambda ^2} - 0.03931}} - 0.00437{\lambda ^2}.\end{equation*}

The CLM crystals of size 4 × 5 × 25 and 4 × 5 × 30 mm^3^ (Fig. 3b), polished to an optical grade on the end faces (4 × 5 mm^2^) and coated with conducting resin on the opposite sides (5 × 25/30 mm^2^) as the E-O components in given crystal orientations (Fig. 3c), were adopted to measure the E-O coefficient using the traditional half-wave voltage method (shown in the ‘Methods’ section); the experimental device and optical path are schematically displayed in Fig. 3d. The E-O coefficient of CLM at different wavelengths was figured out using the refractive indices and half-wave voltage according to Equation (18) in the ‘Methods’ section and the results are listed in Table 3 and illustrated in Fig. 3e and f. The measured E-O coefficient of CLM was ∼10.71 pm V^–^^1^ at the wavelengths varying from the visible to NIR regions, which was close to the calculated value through our powder method. As a comparison, the half-wave voltages and the E-O coefficients of KDP at different wavelengths consulted from *NonlinearOptical Crystals: A Complete Survey* are displayed together in Fig. 3e and f [33]. Overall, within a wavelength region, the CLM exhibited comparable E-O coefficients to KDP and a lower half-wave voltage owing to the superiority of the transverse configuration, indicating it as a novel practical E-O crystal. Hence, the powder method proposed in this paper was verified again for the evaluation of E-O crystals efficiently and would promote the discovery of new E-O crystals.

**Table 3.** The half-wave voltages and E-O coefficients of CLM crystals.

Crystals	Wavelength (nm)	Half-wave voltages (V)	E-O coefficients (pm V^–1^)
CLM	532.8	1680	10.49
(4 × 5 × 30 mm^3^)	632.8	1950	10.89
	710.0	2250	10.67
	800.0	2600	10.46
	900.0	3000	10.24
CLM	532.8	1950	10.80
(4 × 5 × 25 mm^3^)	632.8	2250	11.27
	710.0	2650	10.81
	800.0	3000	10.82
	900.0	3450	10.63

## CONCLUSION

In summary, a powder method, combining the powder SHG responses, IRRS and Raman spectrum of the materials in powder form to predict the approximate magnitudes of the E-O coefficients for the evaluation of E-O crystals efficiently, was developed in this paper. The validity of the powder-evaluation method was proved by comparison of the E-O coefficients between the experimental values and the calculated values of numerous reported E-O materials via this method. Also, on account of the preferable calculated E-O coefficient and the relationship between the E-O effect and the macroscopic symmetry of the crystal, CLM was selected as a potential E-O crystal and the high-quality large-sized single crystals were grown using the Czochralski method for E-O coefficient measurement. Ultimately, the calculated E-O coefficient of CLM showed agreement with the experimental results from half-wave voltage measurement, verifying the practicability of the powder method again. This powder method for the evaluation of E-O crystals is not only significant for further understanding of the E-O coefficient, but also has important implications for the high-efficacy screening of promising E-O crystals.

## METHODS

### Synthesis

Powder samples for measurements were synthesized by conventional solid-state reactions in platinum crucibles. A polycrystalline sample of CLM, for example, was synthesized with a stoichiometric mixture of Cs_2_CO_3_, Li_2_CO_3_ and MoO_3_ of 99.99% purity from Adamas. The material was heated in air to 500°C at a rate of 60°C h^–^^1^ and held at this temperature for 2 h to release CO_2_. Then the sample was ground, packed and heated to 700°C at a rate of 50°C h^–^^1^ and kept at this temperature for 2 days. Eventually, the temperature of the material decreased to room temperature. The phase purity of the CLM powder was confirmed using powder X-ray diffraction.

### Powder X-ray diffraction

PXRD analysis was performed using a Miniflex-600 diffractometer with Cu K_α_ (*λ* = 1.540598 Å) radiation in the angular range of 2*θ* = 5–85° at room temperature.

### Spectrum measurement

The IRRS in the 3000–400 cm^–^^1^ range was recorded on a Bruker Optics VERTEX 70 Fourier transform infrared spectrometer using an ATR device. The Raman spectrum in the range of 3000–400 cm^–^^1^ was performed on a Horiba Labram HR800 Evolution Raman spectrometer under a laser excitation at 532 nm.

### Second-harmonic generation

Powder SHG responses were measured using the Kurtz and Perry method with a Q-switched Nd: YAG solid-state laser of fundamental wavelength 1064 nm with frequency doubling at 532 nm. The CLM and ZnO (used as reference) crystals were ground and sieved into the following particle-size range: 25–45, 45–62, 62–75, 75–109, 109–150 and 150–212 μm. The samples were secured in 1-mm-thick plastic holders with an 8-mm-diameter hole.

### Thermal analysis

The thermogravimetric (TG) analysis and differential thermal analysis of CLM were tested using a NETZSCH STA449F3 simultaneous analyser under flowing nitrogen gas. Reference (Al_2_O_3_ crucible) and crystal samples (10 mg) were packed into the same Al_2_O_3_ crucible and heated from 20°C to 900°C at a rate of 10°C min^–^^1^ and then cooled to room temperature at the same rate.

### Crystal growth

A single crystal of CLM was grown from the congruent melt with a stoichiometric molar ratio according to the formula CsLiMoO_4_ by the Czochralski method using a [110] oriented seed. A large Pt crucible (60 mm in diameter × 60 mm in height) loaded with the prepared polycrystalline materials was used to grow the crystals. The crystals were grown in a JGD-600 Czochralski furnace (CETC No. 26 Institute) heated by frequency induction with a water-cooled copper coil. The crucible was surrounded by Al_2_O_3_ insulating materials in order to construct a suitable thermal gradient. During the growth procedure, the pulling speed and the rotation rate ranged from 2.0 to 0.6 mm h^–^^1^ and 5–8 rpm, respectively. After completing the growth, the temperature of the crystals was dropped to room temperature at a rate of 15°C h^–^^1^. The grown CLM crystals were annealed at 700°C to remove strain before cutting.

### Measurement of E-O coefficient

The CLM crystal belonged to point group $\bar{4}3{\rm m}$ in the cubic system with only one non-zero independent component: *γ*_41_. The optical properties of the crystal were isotropic without the electric field. The refractive-index ellipsoid can be expressed as follows:

(14) \begin{equation*}{x^2} + {y^2} + {z^2} = {n^2}.\end{equation*}

With the role of the electric field *E*, the refractive-index ellipsoid was:

(15) \begin{eqnarray*}\frac{{{x^2}}}{{{n^2}}} &+& \frac{{{y^2}}}{{{n^2}}} + \frac{{{z^2}}}{{{n^2}}} + 2{\gamma _{41}}({E_x}yz + {E_y}zx + {E_z}xy)\nonumber\\ && = 1.\end{eqnarray*}

The electric-field direction was designed along the orientation of [001] and the refractive-index ellipsoid would be:

(16) \begin{equation*}\frac{{{x^2}}}{{{n^2}}} + \frac{{{y^2}}}{{{n^2}}} + \frac{{{z^2}}}{{{n^2}}} + 2{\gamma _{41}}{E_z}xy = 1.\end{equation*}

Under the action of the electric field, the optical properties of the crystal changed into those of a biaxial crystal. The angle between the directions of the coordinate system of the refractive-index ellipsoid without the electric field and that in the electric field was 45°. The principal refractive indices were expressed as ${n_{x^{\prime}}} = n + (1/2){n^3}{\gamma _{41}}{E_z}$, ${n_{y^{\prime}}} = n - (1/2){n^3}{\gamma _{41}}{E_z}$ and ${n_{z^{\prime}}} = n$. When the light propagated through the crystal whose length was *L* in the $y^{\prime}$ direction, the phase difference between the component of light in the $x^{\prime}$ direction and that in the $z^{\prime}$ direction was expressed as follows:

(17) \begin{equation*}\Delta \phi = \frac{\pi }{\lambda }\frac{L}{d}{n^3}{\gamma _{41}}U,\end{equation*}

where *L* is the length of the crystal that was propagated by the light, *d* denotes the thickness of the crystal in the electric-field direction and *U* represents the external direct voltage. The refractive indices at different wavelengths can be calculated according to Equation (13). Thus, the E-O coefficient will be obtained if the phase difference can be measured under a certain voltage.

In this work, the E-O coefficient of the CLM crystal was measured using the traditional half-wave-voltage method. A beam under a certain wavelength was propagated through a polarizer. The vibrating direction of the polarizer was in accordance with the principal axis of the refractive-index ellipsoid without an electric field. The beam, after passing through the crystal and an analyser, was captured by an optical-power meter. At the beginning of measurement, the vibrating directions of the polarizer and the analyser were set as the same. Under this circumstance, the power of the beam, captured by an optical-power meter, was maximal. With the increasing voltage, the phase difference became larger and the intensity of the captured beam tended to be weak. When the variation of the phase difference increased to *π*, the beam intensity would be vanishing. At this time, the applied voltage was the half-wave voltage. The E-O coefficient of the CLM crystal can be determined as follows:

(18) \begin{equation*}{\gamma _{41}} = \frac{\lambda }{{{n^3}U}}\frac{d}{L}.\end{equation*}

## Supplementary Material

nwaa104_Supplemental_File
