# Supplementary material for: A powder method for the high-efficacy evaluation of electro-optic crystals
Source: Natl Sci Rev. 2020 May 28;8(3):nwaa104. doi: 10.1093/nsr/nwaa104 (PMC8288434; doi:10.1093/nsr/nwaa104)
Supplement: nwaa104_Supplemental_File [file nwaa104_supplemental_file.docx]

SUPPLEMENTARY DATA

A powder method for the high-efficacy evaluation of electro-optic crystals

Feng Xu^1,2,3^, Ge Zhang^1,3^, Min Luo^1,3^, Guang Peng^1,3^, Yu Chen^1,3^, Tao Yan^1,3,^* and Ning Ye^1,3,^*

^1^Key Laboratory of Optoelectronic Materials Chemistry and Physics, Fujian Institute of Research on the Structure of Matter, Chinese Academy of Sciences, Fuzhou, Fujian 350002, China;

^2^University of the Chinese Academy of Sciences, Beijing 100049, China;

^3^Fujian Science & Technology Innovation Laboratory for Optoelectronic Information of China, Fuzhou, Fujian 350002, China.

*Corresponding author.

E-mail: nye@fjirsm.ac.cn; yantao@fjirsm.ac.cn

Phone: +86-591-63173430

Fax: +86-591-63173437

**SUPPLEMENTARY CALCULATIONS**

The E-O coefficient of KDP is calculated through the phenomenological approach according to equation (12):

$$\gamma=d+\frac{1}{\sqrt{8}}\sum_{r} \frac{M(r)P(r)}{\hbar^{2}\omega_{r}}$$

In this expression, the SHG coefficient *d* is consulted from Nonlinear Optical Crystals: A Complete Survey. The contribution of lattice variation is calculated from IRRS and Raman spectrum.

The function *F*(*ω*) is derived from reflectance *R*(*k*) according to equation (4) to (6). Supplementary Figure 5 shows the fitted curve by Lorentzian function. Then *M*(*r*) can be worked out through equation (7) according to the fitting results listed in Supplementary Table 1.

The function *dS*/*dω* is derived from equation (8) to (10). The fitted curve is shown in Supplementary Figure 6. *P*(*r*) can be worked out through equation (11) using the fitting results listed in Supplementary Table 2. The excitation wavelength of Raman spectrum is 532nm.

The fitted peaks in the same center frequency within a certain range are selected to obtain the contribution of lattice vibration in E-O coefficient (see Supplementary Table 3). Combing *M*(*r*), *P*(*r*) and center frequency *ω_r_*, the contributed value of lattice vibration determined by the data of *γ^o^_r_* is 10.30 pm V^-1^. Summing the SHG coefficient of 0.39 pm V^-1^, the E-O coefficient of KDP is calculated to be 10.69 pm V^-1^ according to equation (12), which is close to the experimental value of 10.50 pm V^-1^.

**SUPPLEMENTARY FIGURES**


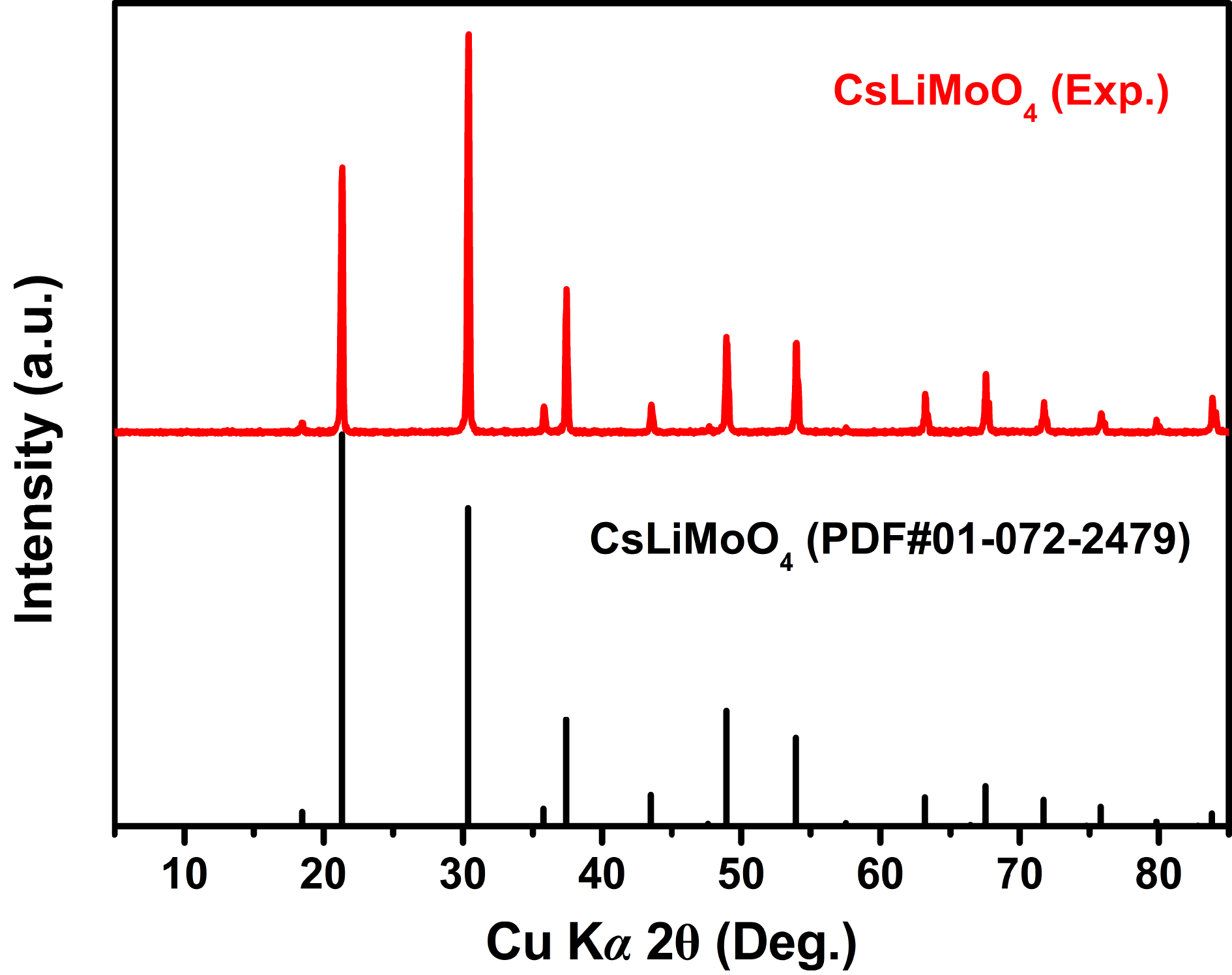


**Supplementary Figure 1.** The powder X-ray diffraction pattern of CLM.

**
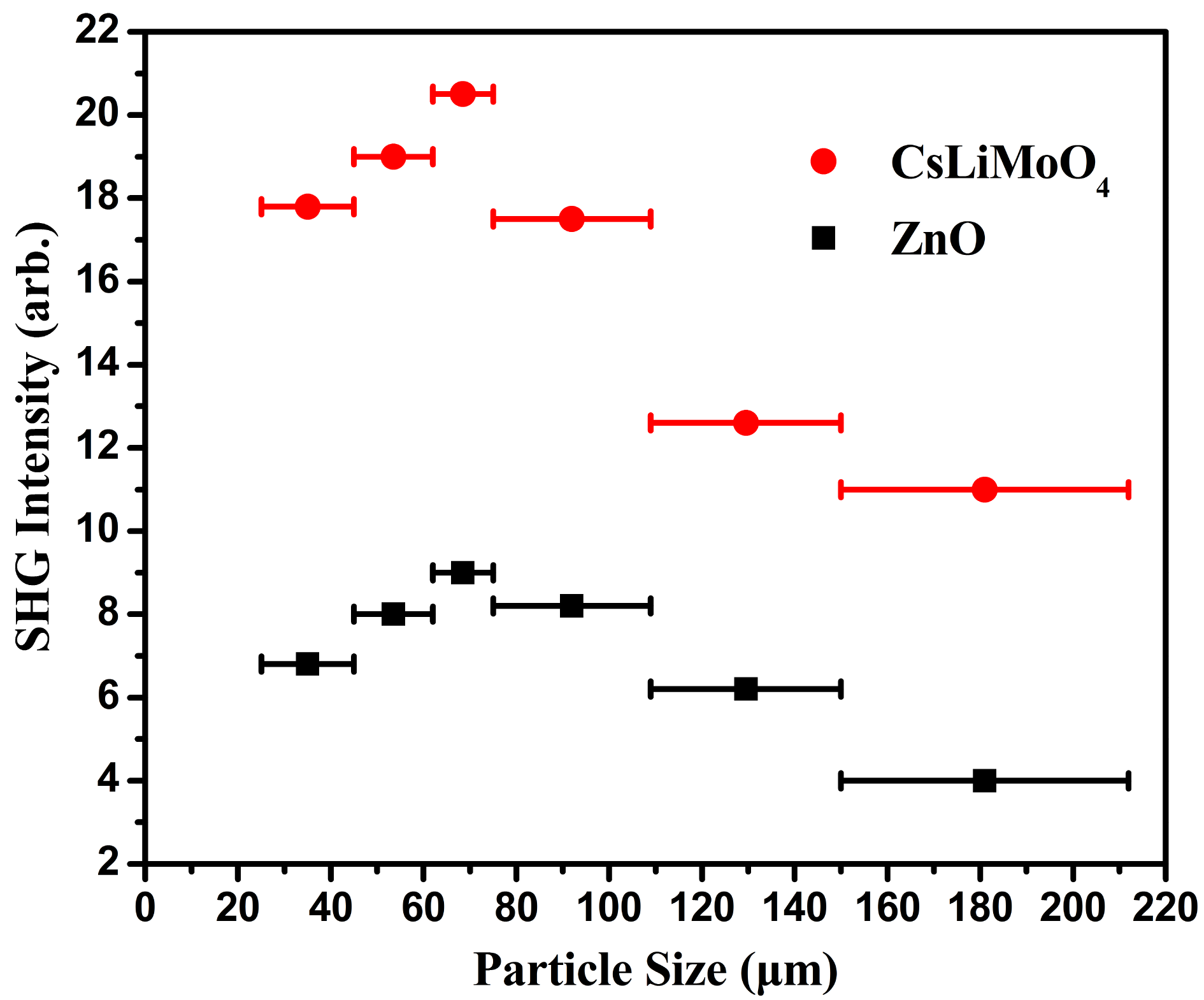
**

**Supplementary Figure 2.** The powder SHG measurement of ground CsLiMoO_4_ (red circle) and ZnO (black square) with the laser at 1064 nm.


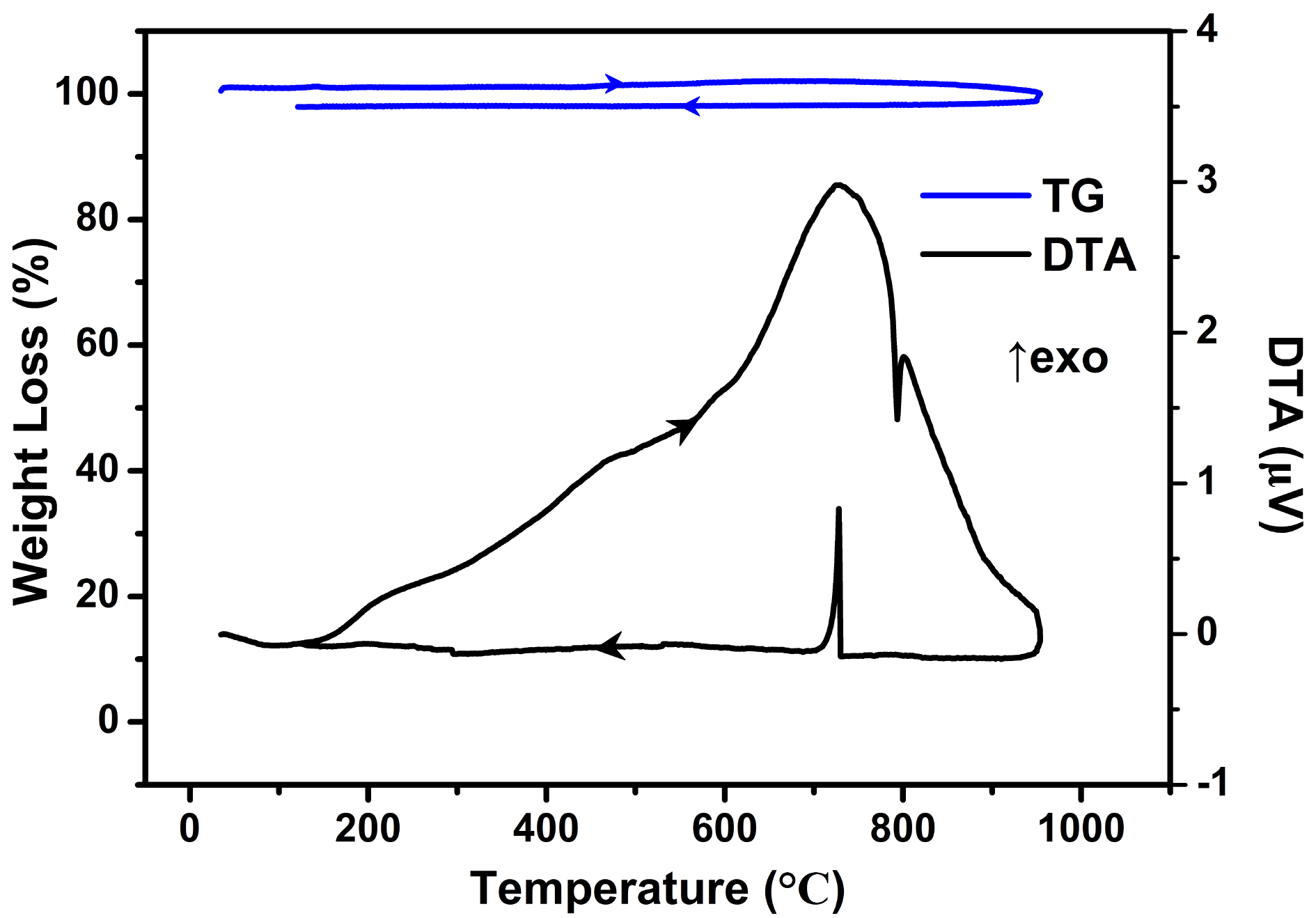


**Supplementary Figure 3.** TG-DTA curves of CLM.


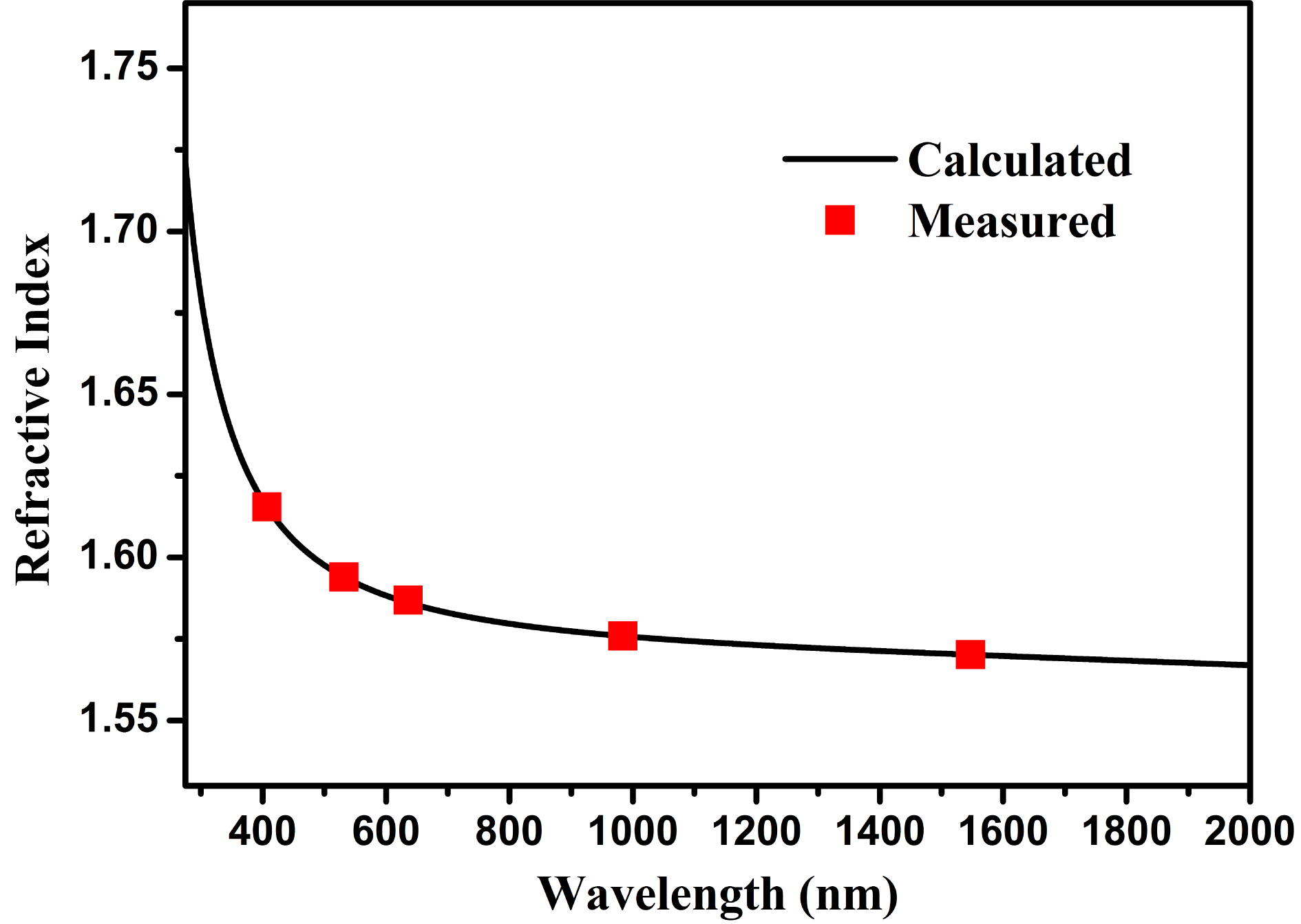


**Supplementary Figure 4.** The measured and fitted refractive indices of CLM crystal.


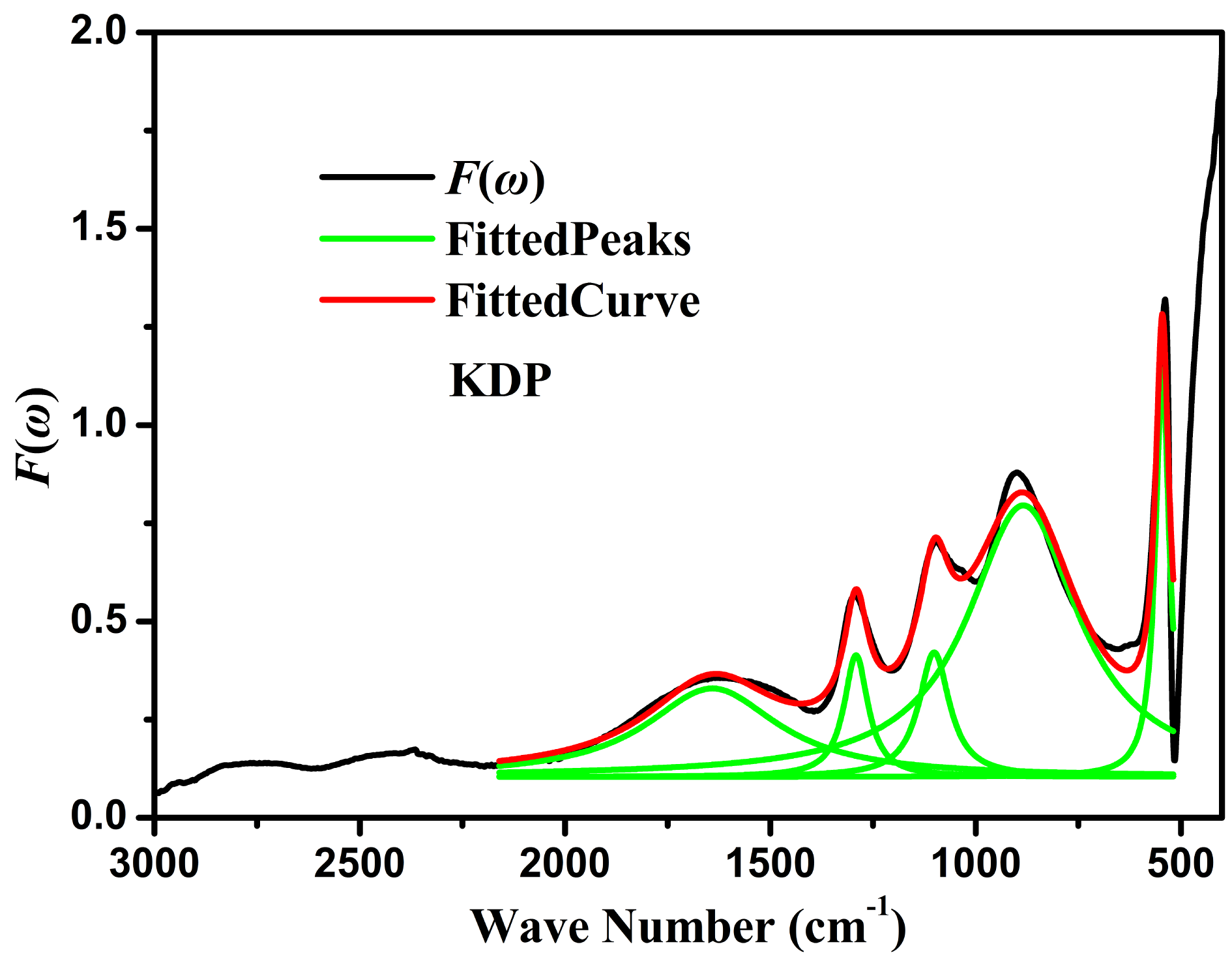


**Supplementary Figure 5.** The *F*(*ω*) of KDP powder according to IRRS.


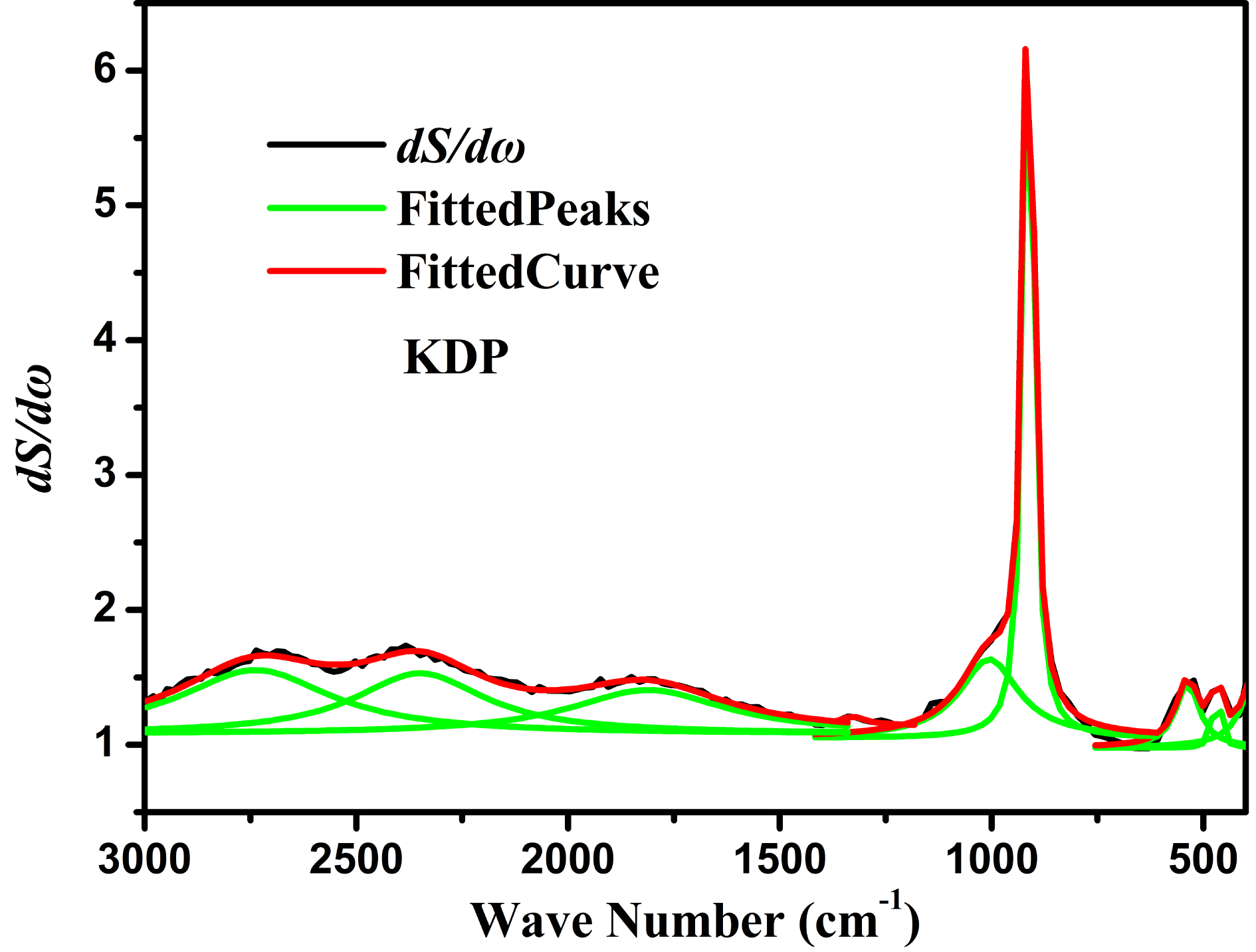


**Supplementary Figure 6.** The *dS*/*dω* of KDP powder according to Raman spectrum.

**SUPPLEMENTARY TABLES**

**Supplementary Table 1.** The curve fitting results of *F*(*ω*) of KDP powder**.**

| Center frequency *ω_i_* (cm^-1^) | Peak width *y_i_* (cm^-1^) | Peak height *H_i_* | *M*(*r*) |
| --- | --- | --- | --- |
| 545.31 | 40.093 | 1.038 | 3.966×10^-12^ |
| 883.94 | 328.582 | 0.692 | 9.268×10^-12^ |
| 1101.08 | 90.609 | 0.317 | 3.297×10^-12^ |
| 1291.28 | 71.136 | 0.310 | 2.887×10^-12^ |
| 1641.52 | 391.917 | 0.225 | 5.777×10^-12^ |

**Supplementary Table 2.** The curve fitting results of *dS*/*dω* of KDP powder.

| Center frequency *ω_i_*  (cm^-1^) | Peak width *y_i_* (cm^-1^) | Peak height *H_i_* | *P*(*r*) |
| --- | --- | --- | --- |
| 468.44 | 11.510 | 1.087 | 1.828×10^-43^ |
| 537.40 | 66.438 | 0.485 | 3.108×10^-43^ |
| 912.32 | 27.442 | 6.433 | 8.926×10^-43^ |
| 1005.75 | 169.172 | 0.586 | 6.926×10^-43^ |
| 1328.22 | 74.477 | 0.065 | 1.678×10^-43^ |
| 1811.48 | 450.510 | 0.327 | 1.009×10^-42^ |
| 2349.90 | 377.271 | 0.451 | 1.153×10^-42^ |
| 2737.73 | 438.350 | 0.475 | 1.314×10^-42^ |

**Supplementary Table 3.** Contributions of lattice vibration in E-O coefficient.

| Center frequency *ω_j_* (cm^-1^) | *M_j_*(*r*) | *P_j_*(*r*) | *γ^o^_r_* |
| --- | --- | --- | --- |
| 537.40 | 3.966×10^-12^ | 3.108×10^-43^ | 6.86 |
| 912.32 | 9.268×10^-12^ | 8.926×10^-43^ | 21.15 |
| 1328.22 | 2.887×10^-12^ | 1.678×10^-43^ | 1.09 |
